# Risk factors for hemodynamic instability during laparoscopic resection of pheochromocytoma

**DOI:** 10.1186/s12894-022-01109-1

**Published:** 2022-09-30

**Authors:** Yong-sheng Huang, Lei Yan, Ze-yan Li, Zhi-qing Fang, Zhao Liu, Zhong-hua Xu, Gang-Li Gu

**Affiliations:** Department of Urology, Qilu Hospital, Shandong University, No 107, Wenhuaxi Road, 250012 Jinan, PR China

**Keywords:** Hemodynamic instability, Laparoscopy, Adrenalectomy, Pheochromocytoma

## Abstract

**Background:**

Laparoscopic adrenalectomy for pheochromocytoma is associated with high risk of intraoperative hemodynamic instability. Our study aimed to identify predictive factors for hemodynamic instability during laparoscopic resection of pheochromocytoma.

**Methods:**

Between January 2011 and December 2021, 136 patients underwent unilateral laparoscopic adrenalectomy for pheochromocytoma. The patients were divided into 2 groups depending on the presence or absence of hemodynamic instability during surgery. Intraoperative hemodynamic parameters were compared between the 2 groups. Patient demographic characteristics and preoperative evaluations were assessed for their prognostic relevance with respect to intraoperative hemodynamic instability via both univariate analysis and multivariate logistic regression analysis.

**Results:**

There was greater blood pressure fluctuations and higher maximum blood pressure and heart rate in the hemodynamic instability group. More patients need intraoperative administration of vasoactive drugs in the hemodynamic instability group. In the univariate analysis, presence of coronary artery disease, tumour size, and previous hypertension history were significantly associated with intraoperative hemodynamic instability. The multivariate logistic regression analysis showed that tumour size and previous hypertension history were independent risk factors for intraoperative hemodynamic instability.

**Conclusion:**

Tumour size and previous hypertension history were associated with hemodynamic instability during laparoscopic resection of pheochromocytoma.

## Background

Adrenal pheochromocytoma derives from catecholamine producing chromaffin cells in the adrenal medulla. Resection of pheochromocytoma carries a high risk of eliciting massive catecholamine release, which can cause severe hypertension [1, 2]. Hypotensive episodes can also occur after tumour resection, requiring the sustained administration of vasopressor drugs. These intraoperative hemodynamic instabilities (HI) can frequently occur even when adequate preoperative medications and hydration have been provided [3, 4]. The widespread experience with mini-invasive surgery have resulted in the establishment of laparoscopic adrenalectomy (LA) as the first choice for adrenal tumours, including pheochromocytoma [5, 6]. So far, few studies focused on LA and its risk factors for intraoperative HI. Therefore, we conducted this study, based on our experience, to identify the possible predictors for HI during laparoscopic resection of pheochromocytoma.

## Methods

The present retrospective study was approved by the institutional review board. 144 pheochromocytoma patients underwent unilateral LA via transperitoneal or retroperitoneal approach from January 2011 and December 2021. 2 patients with history of homolateral abdominal surgery and 6 patients undergoing conversion to laparotomy were excluded, leaving 136 patients enrolled in the study. Patients were divided into 2 groups depending on presence or absence of intraoperative HI. As previously described [7], HI was defined as an occurrence of intraoperative episodes of systolic blood pressure (SBP) above 160 mmHg and vasoactive drug administration.

The preoperative diagnosis of pheochromocytoma was based on clinical manifestations and computed tomography or magnetic resonance image findings. All patients suspected of pheochromocytoma were prepared for surgery by administration of phenoxybenzamine for at least 2 weeks to normalize blood pressure (BP) (＜140/90 mmHg), irrespective of previous hypertension history. Beta-blockers was added to control tachyarrhythmia if necessary. Adequate hydration was administered to all patients preoperatively. The final diagnosis of pheochromocytoma was confirmed by postoperative pathology.

Transperitoneal or retroperitoneal LA was performed by surgeons expertizing in adrenal surgery (> 50 cases per surgeon). Four surgeons preferred transperitoneal approach, whereas one surgeon preferred retroperitoneal approach.

For transperitoneal LA, patients were placed in semi-lateral decubitus positions with the side of the lesion elevated at 70°. Pneumoperitoneum with a pressure of 12 mmHg was established using a Veress needle through a 10-mm supraumbilical incision. A 10-mm trocar was introduced via the incision to serve as camera port. Two subcostal trocars were placed in the midclavicular and anterior axillary lines, respectively. For right-sided procedures, an additional trocar was placed below the xiphoid for liver traction. An incision on the posterior parietal peritoneum above the descending segment of duodenum was made longitudinally along the right margin of vena cava and subsequently extended outward along the reflection of the peritoneum and liver. Attachments between the adrenal gland and the liver were divided. The vena cava was separated from the adrenal gland to expose the medial adrenal border, through which an aspirator was inserted into the space between the gland and diaphragm to retract the gland laterally. The arterioles along the medial border were divided until the right adrenal vein was identified and divided. Attachments along the upper adrenal border were subsequently divided. Finally, the gland was separated from the upper pole of the kidney and resected. For left-sided procedures, an incision of the spleno-diaphragmatic ligaments was made deep enough to visualize the gastric fundus and the left diaphragmatic crus. The spleno-pancreatic bloc was mobilized medially to expose the adrenal region. The left renal vein was identified and an aspirator was inserted between the adrenal gland and the renal vein to retract the tumour upward. Attachments along the lower adrenal border were divided until the adrenal vein was identified and divided. The attachments along the medial border were divided. Finally, the adrenal gland was separated from the upper pole of the kidney and resected.

For retroperitoneal LA, patients were placed in lateral decubitus positions. Operating table was flexed to expand the space between the 12th rib and the iliac crest. A 15-mm incision was made in the midaxillary line, 1 cm above the iliac crest. The retroperitoneal space was initially created using the index finger of the surgeon and then expanded using a self-made dissector inflated with 700 ml of air. A 10-mm trocar was introduced through the incision to serve as camera port. Two operating trocars were placed below the costal margin in the anterior axillary and the posterior axillary lines, respectively. Gerota’s fascia was incised. Dissection was initially performed between the perirenal fat and anterior renal fascia, and subsequently along the psoas muscle to expose the adrenal gland. The gland was separated from the upper pole of the kidney until the adrenal vein was identified and divided. Finally, attachments of the upper and medial adrenal borders were divided and the tumour was resected.

Intraoperative hemodynamic parameters were compared between the HI and non-HI groups. Demographic characteristics of the patients and preoperative evaluations were assessed for their prognostic relevance for intraoperative HI. The variables considered were age, sex, body mass index (BMI), presence or absence of preoperative symptoms, comorbidities, smoking and drinking, tumour laterality, tumour size, surgical approach, previous hypertension history, duration of hypertension, family history of hypertension and BP after alpha-blockade. Hypertension was defined as SBP > 140 mmHg or diastolic blood pressure (DBP) > 90 mmHg.

Statistical analyses were conducted to determine the prognostic relevance of preoperative factors for HI. Parametric data (presented as mean ± standard) were compared using t-test. Non-parametric data (presented as median and min–max range) were compared using Mann-Whitney U test. Categorical data were compared using χ2 test or Fisher’s exact test. A multivariate logistic regression analysis was conducted to determine the independent risk factors for HI. All p-values were two-sided and p < 0.05 was considered significant. Analyses were conducted using SPSS 25.0 (SPSS Inc., Chicago, IL, USA).

## Results

The HI group and non-HI group included 84 and 52 patients, respectively. Table 1 presents the intraoperative hemodynamic parameters of these 2 groups. The highest intraoperative SBP (180.74 ± 14.41 vs. 147.62 ± 13.85 mmHg, p = 0.000) and DBP (106.89 ± 12.84 vs. 92.17 ± 11.33 mmHg, p = 0.000), incidence of SBP ≥ 200 mmHg (10.71% vs. 0%, p = 0.013) and highest intraoperative heart rates (HR) (102.69 ± 15.62 vs. 93.90 ± 15.02 beat/min, p = 0.002) were significantly higher in the HI group. The difference between the 2 groups in incidence of highest HR ≥ 110 bpm was marginally significant (34.52% vs. 19.23%, p = 0.055). The differences between the highest and lowest SBP and DBP, which reflected intraoperative BP fluctuation, were significantly higher in the HI group (maximal difference of SBP [78.88 ± 17.08 vs. 46.40 ± 15.85 mmHg, p = 0.000], maximal difference of DBP [45.24 ± 10.61 vs. 30.38 ± 12.11 mmHg, P = 0.000], respectively). More patients need intraoperative vasoactive drugs administration in the HI group (vasodilator drugs [69.05% vs. 15.38%, p = 0.000], vasoconstrictive drugs [17.86% vs. 1.92%, p = 0.005], beta-blockers [78.57% vs. 55.77%, p = 0.005], respectively). Intraoperative injury of short hepatic vein occurred in 1 patient of the HI group. Postoperative hypotension occurred in 2 patients of the HI group, which required sustained vasopressor support. No perioperative death occurred in our cohort.

**Table 1:** Intraoperative hemodynamic parameters of HI group vs. Non-HI group.

	HI group(***n*** = 84)	Non-HI group(***n*** = 52)	***P*** value
Highest intraoperative SBP(mmHg)	180.74 ± 14.41	147.62 ± 13.85	0.000
Lowest intraoperative SBP(mmHg)	101.86 ± 14.57	101.31 ± 13,0.47	0.826
Highest intraoperative DBP(mmHg)	106.89 ± 12.84	92.17 ± 11.33	0.000
Lowest intraoperative DBP(mmHg)	61.77 ± 10.05	61.60 ± 11.72	0.925
Incidence of SBP ≥ 200 mmHg(n)	9(10.71%)	0(0%)	0.013*
Incidence of SBP < 80 mmHg(n)	4(4.76%)	4(7.69%)	0.481*
Highest heart rate(beat/min)	102.69 ± 15.62	93.90 ± 15.02	0.002
Lowest heart rate(beat/min)	59.57 ± 12.20	59.87 ± 11.66	0.890
Incidence of highest HR ≥ 110 bpm(n)	29(34.52%)	10(19.23%)	0.055
Incidence of lowest HR < 50 bpm(n)	14(16.67%)	7(13.46%)	0.615
Highest SBP-lowest SBP(mmHg)	78.88 ± 17.08	46.40 ± 15.85	0.000
Highest DBP-lowest DBP(mmHg)	45.24 ± 10.61	30.38 ± 12.11	0.000
Patients requiring intraoperative vasodilator drugs(%)	58(69.05%)	8(15.38%)	0.000
Patients requiring intraoperative vasoconstrictive drugs(%)	15(17.86%)	1(1.92%)	0.005
Patients requiring intraoperative beta-blockers(%)	66(78.57%)	29(55.77%)	0.005

Footnotes: SBP Systolic blood pressure, DBP Diastolic blood pressure, HR Heart rate, *Fisher’s Exact Test

Patient characteristics of both groups were listed in Table 2. The difference in age between HI and non-HI group was marginally significant (47.5 ± 14.1 vs. 42.6 ± 14.1, p = 0.051). Presence of coronary artery disease was more common in HI group (17.9% vs. 3.8%, p = 0.016). The tumour size of the HI group was significantly larger (50 [15–110] vs. 41 [7-110] mm, p = 0.004). More patients had previous hypertension history in HI group (69% vs. 44.2%, p = 0.004). Patient characteristics such as BMI, sex, presence of preoperative symptoms, presence of diabetes mellitus and cerebrovascular disease, tumour laterality, surgical approach, duration of hypertension, family history of hypertension, history of smoking and drinking, and BP after alpha-blockade did not differ statistically between the 2 groups.

**Table 2:** Characteristics of pheochromocytoma patients in the HI and non-HI groups

	HI group (n = 84)	Non-HI group(n = 52)	p-value
Age	47.5 ± 14.1	42.6 ± 14.1	0.051
BMI	22.68 ± 3.62	22.21 ± 2.70	0.393
Sex			0.579
Male	38(45.2%)	21(40.4%)	
Female	46(54.8%)	31(59.6%)	
Presence of Preoperative symptoms	52(61.9%)	36(69.2%)	0.385
Comorbidities			
Diabetes mellitus	20(23.8%)	9(17.3%)	0.368
Coronary artery disease	15(17.9%)	2(3.8%)	0.016
Cerebrovascular disease or accident	9(10.7%)	2(3.8%)	0.134*
Tumour laterality			0.568
Right	41(48.8%)	28(53.8%)	
Left	43(51.2%)	24(46.2%)	
Tumour size(mm)	50(15–110)	41(7−110)	0.004
Surgical approach			0.424
Transperitoneal	75(89.3%)	44(84.6%)	
Retroperitoneal	9(10.7%)	8(15.4%)	
Previous hypertension history	58(69.0%)	23(44.2%)	0.004
Duration of hypertension (mo)	36(1−480)	24(1−240)	0.809
Family history of hypertension	2(2.4%)	3(5.8%)	0.370
Smoking history	9(10.7%)	4(7.7%)	0.766*
Drinking history	9(10.7%)	4(7.7%)	0.766*
SBP (mmHg) after alpha-blockade	134.94 ± 20.98	130.46 ± 22.23	0.239
DBP (mmHg) after alpha-blockade	82.93 ± 15.17	78.67 ± 13.75	0.102

Footnotes: BMI body mass index, SBP Systolic blood pressure, DBP Diastolic blood pressure, *Fisher’s exact test

Of the investigated risk factors, presence of coronary artery disease, tumour size and previous hypertension history were included in multivariate logistic regression analysis. Age was also included in the multivariate analysis, considering the difference between the 2 groups was marginally significant. The result revealed that tumour size (OR 1.029, 95% CI: 1.010–1.049, P = 0.002) and previous hypertension history (OR 3.044, 95% CI: 1.384–6.697, P = 0.006) were independent risk factors associated with intraoperative HI (Table 3).

**Table 3:** Risk factors for HI in patients undergoing laparoscopic adrenalectomy for pheochromocytoma (multivariate logistic regression analysis)

Variable	Odds ratio	95% CI		p-value
		Lower	Upper	
Age	1.015	0.987	1.043	0.301
Presence of coronary artery disease	3.818	0.753	19.347	0.106
Tumour size(mm)	1.029	1.010	1.049	0.002
Previous hypertension history	3.044	1.384	6.697	0.006

Footnotes: CI, confidence interval

## Discussion

HI during surgical resection of pheochromocytomais is still a big concern for surgeons and anesthesiologists, since it has been proven to be associated with morbidity [8, 9]. However, there was still no agreement on the definition of intraoperative HI according to the studies. Bruynzeel and Brunaud indicated that intraoperative SBP above 160 mmHg was a risk factor for HI [2, 8] and Aksakal et al. revealed that HI was associated with the requirement of cardioactive or vasoactive drugs administration [10]. Integrating those findings, Pisarska-Adamczyk et al. defined HI as an occurrence of both intraoperative episodes of SBP above 160 mmHg and requirement of vasoactive drug administration [7]. In our study, based on this definition, the hemodynamic parameters including the highest intraoperative BP, incidence of extreme hypertensive episodes, the highest HR and the range of BP fluctuation were significantly higher in the HI group, indicating it could objectively reflect the intraoperative hemodynamic status. The incidence of intraoperative HI was 61.8% in our series, which was higher than previous reports [7, 10]. However, considering the heterogeneity of HI definition, operating techniques, tumour size and perioperative patient management, it is difficult to compare the results among different studies.

Nowadays LA is the gold standard of surgical treatment for adrenal tumours. For pheochromocytoma, there has been debate in the literature whether the formation of pneumoperitoneum could lead to a massive release of catecholamines, resulting in hypertension and tachycardia [11]. However, several recent studies have demonstrated that LA is equally safe as open surgery and does not increase the risk of HI [12, 13]. Therefore, LA gradually became the first choice for resection of pheochromocytoma, especially for small to medium-sized tumour. As LA became more technologically mature, its indication was extended to large tumours [14]. In the present study, the maximal diameter of the tumours was 110 mm for both transperitoneal and retroperitoneal approaches. To our knowledge, only few studies specially focused on LA and its predictors for intraoperative HI, and moreover, the tumour was confined to medium size [7].

Similar to the results of the previous studies [3, 10], we demonstrated that tumour size was an independent risk factor for intraoperative HI. Larger tumours have a tendency to secrete higher levels of catecholamines and as a result to increase the incidence and duration of intraoperative hypertensive episodes [2, 15, 16]. In addition, larger tumours required increased manipulation during tumour dissection and resection, resulting in high catecholamine release and significant hemodynamic fluctuation [17].

Some underlying diseases can impair the cardiovascular function, which may in turn influence intraoperative hemodynamic parameters. Pisarska-Adamczyk et al. [7] and Bai et al. [18] demonstrated that presence of diabetes mellitus and coronary artery disease were independent risk factors for HI, respectively. In the present study, however, we did not prove a causal link between patients’ comorbidities and HI. The incidence of diabetes mellitus in the HI and non-HI group was comparable. Although coronary artery disease was significantly more common in the HI group, it was not proven to be associated with the development of HI in the multivariate logistic regression analysis.

Our study showed that previous hypertension history was an independent risk factor for intraoperative HI. As is known, a proportion of patients with pheochromocytoma are normotensive. Normotension is seen mostly in patients with relatively small amounts of catecholamines in circulation. On the other hand, sustained and paroxysmal hypertension strongly correlates with high levels of plasma norepinephrine and epinephrine, respectively [19]. Increased plasma level of catecholamine and/or metabolites has been confirmed to be associated with intraoperative HI [20]. That may explain why patients with previous hypertension history were more prone to intraoperative HI. Furthermore, hypertensive patients have higher arterial stiffness compared to normotensive ones, so these patients may be relatively susceptible to hypertension episodes.

With respect to LA for pheochromocytoma, only a few studies have evaluated the effects of surgical approach on the occurrence of HI with conflicting results. Vorselaars et al. showed that retroperitoneal approach carried greater risk of intraoperative hypotension than transperitoneal approach [9]. On the contrary, Ban et al. found retroperitoneal approach provided favorable intraoperative hemodynamic parameters compared with transperitoneal approach [21]. In their opinion, the anatomical benefits of retroperitoneal approach enables minimum manipulation of the tumour and early control of the adrenal vein, which reduces excessive catecholamine secretion. However, when performing retroperitoneal LA, we found it was sometimes difficult to access the adrenal vein underneath larger tumours. Moreover, the right kidney often blocks the way to control the adrenal vein, making early ligation of the adrenal vein more difficult. On the other hand, when performing transperitoneal LA, we initially placed an aspirator between the adrenal gland and vena cava or left renal vein to retract the tumour. In this way, attachments along the border of the glands and near the adrenal vein could be clearly exposed and divided. This technique allows the surgeons to isolate and control the adrenal vein at the early stage of surgery via transperitoneal approach. In the present study, we found surgical approach had no significant impact on intraoperative HI. This was consistent with the findings of a recent meta-analysis by Jiang et al. [22], which demonstrated no significant difference between transperitoneal and retroperitoneal approaches in the incidence of hemodynamic crisis, though some advantages for retroperitoneal approach including shorter operative time and less blood loss were reported.

Numerous other risk factors for HI were reported in the literature, including plasma or urinary level of catecholamine, BMI, clinical symptoms and et al. [3, 9, 23, 24]. Possible reasons for this variability might be a lack of standardized perioperative anaesthesiological and surgical management. In addition, discrepancies could arise from different HI definitions. Among the possible risk factors, plasma or urinary level of catecholamine has been most frequently reported [24]. Unfortunately, we did not routinely test that before the year of 2020 in our center. Thus, preoperative levels of catecholamine were not evaluated in the current study.

This study has limitations typical of a single center study. A relatively limited number of patients were included, mainly due to the rare occurrence of pheochromocytoma. Second, this is a retrospective study, and selection bias was not avoidable. Another important limitation of the study seems to be the choice of the definition of HI. The multitude of definitions presented by various authors makes it difficult to compare the results.

## Conclusion

Overall, in our group, hemodynamic instability occurred in more than half of the patients undergoing LA for pheochromocytoma. The size of the adrenal tumour and previous hypertension history were independent risk factors of HI during laparoscopic resection of pheochromocytoma.

## Data Availability

The datasets used and analyzed during the current study are available from the corresponding author on reasonable request.

## List of abbreviations

HI: hemodynamic instabilities.
LA: laparoscopic adrenalectomy.
SBP: systolic blood pressure.
BP: blood pressure.
BMI: body mass index.
DBP: diastolic blood pressure.
HR: heart rates.
